# Molecular Characterization of Presumptive *Klebsiella pneumoniae* Isolates from Companion and Farm Animals in Germany Reveals Novel Sequence Types

**DOI:** 10.3390/pathogens14030259

**Published:** 2025-03-05

**Authors:** Marwa Bassiouny, Peter A. Kopp, Ivonne Stamm, Hanka Brangsch, Heinrich Neubauer, Lisa D. Sprague

**Affiliations:** 1 Institute of Bacterial Infections and Zoonoses, Friedrich-Loeffler-Institut (FLI), 07743 Jena, Germany; 2 Institute for Pharmacy, Friedrich Schiller University Jena, 07745 Jena, Germany; 3 Vet Med Labor GmbH, IDEXX Laboratories, Humboldtstrasse 2, 70806 Kornwestheim, Germany

**Keywords:** *Klebsiella pneumoniae*, companion and farm animals, MLST, WGS, novel sequence types, Germany

## Abstract

*Klebsiella* (*K*.) *pneumoniae* is a One Health pathogen that has been isolated from humans, animals, and environmental sources and is responsible for a diverse range of potentially life-threatening infections. In the present study, we analyzed the genomes of 64 presumptive *K. pneumoniae* strains isolated in 2023 from different companion and farm animals in Germany. Using whole-genome sequencing (WGS) data, 59 isolates (92.2%) were identified as *K. pneumoniae* and five (7.8%) as *K. quasipneumoniae*. Multilocus sequence typing (MLST) assigned 53 isolates to 46 distinct sequence types (STs). Eleven isolates could not be assigned to existing STs of the Pasteur classification scheme because they contained novel alleles not previously documented. Thus, these were considered novel and designated as ST7681-ST7689 and ST7697-ST7698. Almost all isolates in this study were assigned unique STs, and only five STs were shared among multiple isolates. This research highlights the genetic diversity among *K. pneumoniae* strains isolated from different companion and farm animals in Germany, provides information to help in surveillance strategies to mitigate zoonotic transmission risks, and demonstrates the value of WGS and MLST in identifying novel STs of *K. pneumoniae*.

## 1. Introduction

*Klebsiella* (*K*.) *pneumoniae* is a Gram-negative, non-motile, encapsulated, and facultatively anaerobic bacterium belonging to the family Enterobacteriaceae [1]. It is ubiquitous in nature and can be found in animals, water, and soil [2]. *K. pneumoniae* is an opportunistic pathogen recognized globally as one of the most critical multidrug-resistant (MDR) microorganisms [3]. It is a leading cause of hospital-acquired infections worldwide [4]. It can cause serious diseases, including pneumonia and urinary tract and bloodstream infections, as well as liver abscesses [2], with high mortality rates due to its resistance to multiple antibiotics [1]. *K. pneumoniae* is characterized by various virulence factors that contribute to its pathogenicity. These include a polysaccharide capsule, surface lipopolysaccharides, fimbriae, and siderophores, which facilitate adhesion to host tissues, evasion of the immune response, and acquisition of essential nutrients [5]. Additionally, it possesses the ability to form biofilms and harbors a diverse array of resistance genes, enhancing its resilience against aminoglycosides, quinolones, polymyxins, and β-lactams [6]. In animals, *K. pneumoniae* is highly pathogenic and can affect the urogenital, respiratory, and digestive systems [7]. Its ability to infect nearly every organ or tissue makes *K. pneumoniae* a significant concern in animal health [8]. In Germany, multiple outbreaks of *K. pneumoniae* have been reported in humans [9,10], and it has been isolated from various animals such as dogs, cats, horses, pigs, and cattle [11,12,13,14] as well as from milk powder [15]. However, the knowledge on STs of *K. pneumoniae* is limited in Germany, due to a lack of isolated strains. This study aimed to perform molecular characterization of 64 presumptive *K. pneumoniae* isolates from companion and farm animals across various federal states in Germany, and to identify novel STs.

## 2. Materials and Methods

### 2.1. Bacterial Isolates and Identification

Sixty-four presumptive *K. pneumoniae* isolates from the strain collection of IDEXX Laboratories, Kornwestheim, Germany, were used in the current study. The isolates were isolated from various companion and farm animals in Germany in 2023. The majority of isolates (75%, 48/64) were obtained from dogs, followed by horses (17.2%, 11/64), cats (4.7%, 3/64), and 1.6% (1/64) each from cattle and chickens, as shown in Table 1. All isolates were identified at the species level using Matrix-Assisted Laser Desorption/Ionization Time-of-Flight Mass Spectrometry (MALDI-TOF MS). Sample preparation, protein extraction, and species identification using MALDI-TOF were conducted as previously described [16] using a Microflex LT instrument (Bruker Daltonics, Bremen, Germany).

### 2.2. DNA Extraction, WGS, and In Silico Detection of Sequence Types

Genomic DNA extraction was performed from a single colony grown overnight on Columbia blood agar at 37 °C using the High Pure PCR Template Preparation Kit (Roche Diagnostics GmbH, Mannheim, Germany) according to the manufacturer’s instructions. Nextera XT DNA Library Preparation Kit was used to prepare sequencing libraries, and paired-end sequencing was carried out on an Illumina MiSeq sequencer (Illumina Inc., San Diego, CA, USA). Raw sequencing data analysis and quality checks of the assembled genomes were performed as previously described [17,18]. The multilocus sequence typing (MLST) was determined in silico using the in-house pipeline WGSBAC (https://gitlab.com/FLI_Bioinfo/WGSBAC, accessed on 2 December 2024) and the software mlst v2.16.1 (https://github.com/tseemann/mlst, accessed on 2 December 2024) that uses the PubMLST website [19] and the scheme proposed by Diancourt and colleagues [20], known as the Pasteur typing scheme. Neighbor-Joining (NJ) analysis was performed using GrapeTree software for constructing a phylogenetic tree [21]. Microreact was employed to visualize both epidemiological data and phylogenetic trees [22].

## 3. Results

### 3.1. Bacterial Isolate Identification and MLST Analysis

**Figure 1:**
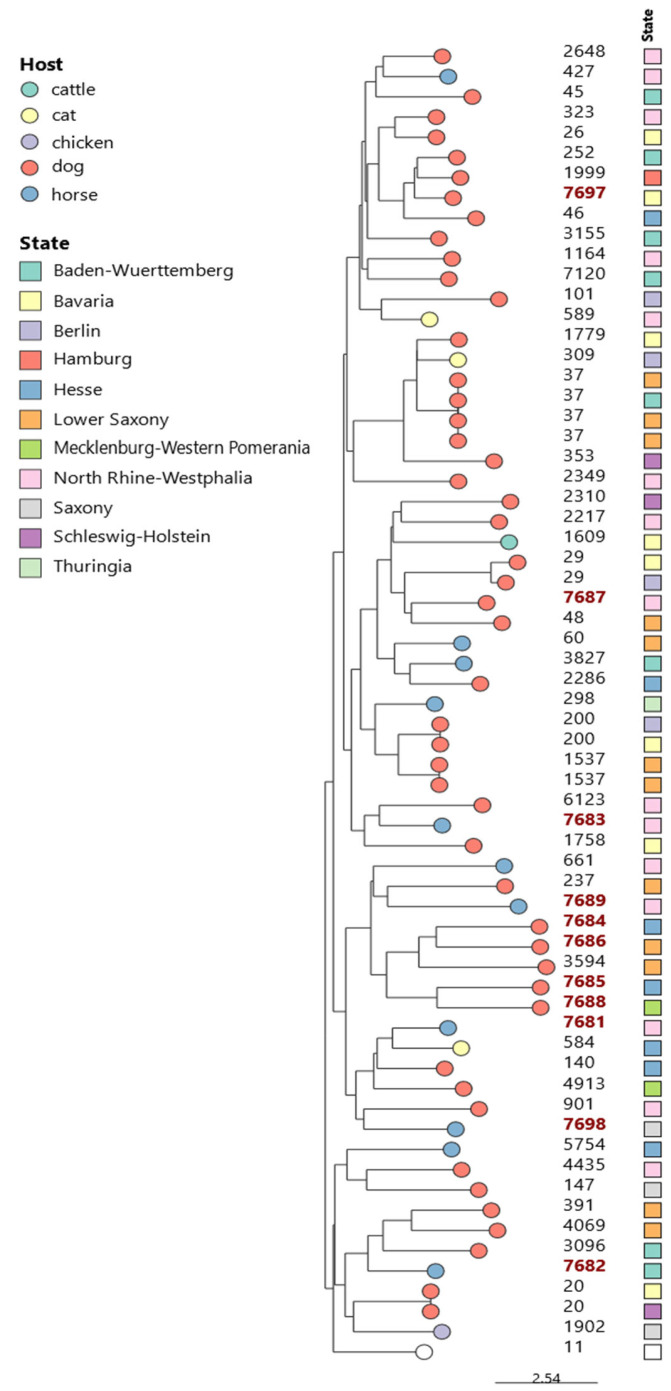
The phylogenetic tree of 64 *K. pneumoniae*/*quasipneumoniae* isolates from companion and farm animals in Germany was constructed using the Neighbor-Joining (NJ) method based on MLST data. The tree includes STs and corresponding geographical locations. Novel STs are indicated in red.

MALDI-TOF MS initially identified all 64 *K. pneumoniae* isolates as *K. pneumoniae*. However, subsequent WGS-based analysis only identified 59 strains (92.2%) as *K. pneumoniae* and 5 (7.8%) as *K. quasipneumoniae*. MLST analysis revealed that the majority of the strains (53/64, 82.8%) belonged to 46 distinct STs, as shown in Table 1 and Figure 1.

### 3.2. Novel STs, Their Hosts, and Geographical Distribution

Of the 64 studied strains, 17.2% (*n* = 11) could not be assigned to any known ST and were therefore classified as novel STs according to the institute Pasteur database (https://bigsdb.pasteur.fr/, accessed on 29 October 2024). Seven of them were identified as *K. pneumoniae* and four as *K. quasipneumoniae*. The newly identified STs included ST7681 to ST7689 and ST7697 to ST7698. These novel STs were detected in isolates from dogs (*n* = 6) and horses (*n* = 5), originating from various sample materials, including the feces, uterus, cervix, and urine. The isolates were distributed across seven different federal states, as shown in Figure 1 and Table 2.

## 4. Discussion

In the present study, we characterized 64 presumptive *K. pneumoniae* strains isolated from different companion and farm animal species in Germany in 2023. These isolates were initially identified as *K. pneumoniae* using MALDI-TOF MS. However, subsequent confirmation via whole-genome sequencing (WGS) revealed that 59 isolates were *K. pneumoniae* and 5 were *K. quasipneumoniae*. The five *K. quasipneumoniae* strains were found in fecal and urine samples from dogs. *K. quasipneumoniae* was described for the first time in 2014 and identified in human infections [23]. *K. quasipneumoniae* has been isolated from humans, animals, and various environmental sources in Germany [24,25,26], as well as in other European countries, including France [23], Sweden [27], Portugal [28], and Italy [29]. Our study also shows a genetic diversity, with 57 sequence types (STs) among 64 *K. pneumoniae*/*quasipneumoniae* strains. Altogether, 52 STs were represented by a single isolate each, including eleven novel STs. These novel STs originated from seven different German federal states, highlighting their geographical spread. Detecting novel alleles is essential for advancing future surveillance and diagnostic strategies. Some identified STs in this study, such as ST45, ST29, ST101, and ST147, have been previously reported in human infections in Germany [30,31,32,33], highlighting the potential for zoonotic transmission of *K. pneumoniae*.

In conclusion, collaborative efforts between veterinary and public health sectors are necessary to improve our understanding of transmission dynamics between companion and farm animals and humans. The findings of this study highlight the importance of molecular examination of *Klebsiella* strains isolated from animals, as it enables the identification of novel sequence types, reveals genetic diversity, and provides insights into their epidemiological significance. Further molecular studies on isolates from different one-health sectors are necessary to assess transmission pathways and the epidemiological impact of *K. pneumoniae* or *K. quasipneumoniae*.

## Figures and Tables

**Table 1:** Host, source, and MLST diversity of 53 *K. pneumoniae*/*quasipneumoniae* strains with 46 distinct STs obtained from various companion and farm animals in Germany in 2023.

Host	Sample Origin	Number of Isolates	MLST
Dogs (*n* = 42)	Feces	27	1537 *, 200 *, 37 **, 101, 29, 1999, 7120, 2349, 3155, 46, 1164, 4435, 45, 2648, 3096, 323, 1779, 3594 ^#^, 2217, 2286, 26, 20, 252
	Urine	2	4069, 1758
	Tracheal swab	3	2310, 140, 237
	Wound	2	353, 391
	Nose	1	48
	Skin	1	37
	Eye	1	147
	Vocal cords	1	6123
	Abdominal abscess	1	4913
	Intestine	1	29
	Uterus	1	20
	ND	1	901
Horses (*n* = 6)	Nose	2	3827, 661
	Wound	1	60
	Cervix	1	427
	Penis	1	298
	Uterus	1	5754
Cats (*n* = 3)	Feces	2	309, 589
	Ear	1	584
Cattle (*n* = 1)	Nose	1	1609
Chicken (*n* = 1)	Feces	1	1902

ND: Not determined, *: was identified in 2 isolates, ** was identified in 3 isolates, ^#^ *K. quasipneumoniae* isolate. *n*: Number of isolates; MLST: multilocus sequence typing.

**Table 2:** MLST characteristics of the eleven *K. pneumoniae*/*quasipneumoniae* isolates with novel STs obtained from companion and farm animals in Germany in 2023.

ST	MLST Profile							*Klebsiella* Species	Sample Origin	Source	Geographical Origin
	*gapA*	*infB*	*mdh*	*pgi*	*phoE*	*rpoB*	*tonB*				
7681	4	1	2	1	1	4	61	*K. pneumoniae*	Feces	Horse	North Rhine-Westphalia
7682	1	1	1	1	1	4	4	*K. pneumoniae*	Uterus	Horse	Baden-Wuerttemberg
7683	2	1	37	1	9	1	31	*K. pneumoniae*	Cervix	Horse	North Rhine-Westphalia
7684	18	22	327	223	11	105	99	*K. quasipneumoniae*	Feces	Dog	Hesse
7685	17	55	73	20	103	18	608	*K. quasipneumoniae*	Feces	Dog	Hesse
7686	18	22	55	22	193	54	50	*K. quasipneumoniae*	Feces	Dog	Lower Saxony
7687	2	1	2	2	7	4	23	*K. pneumoniae*	Urine	Dog	North Rhine-Westphalia
7688	17	80	92	306	100	18	162	*K. quasipneumoniae*	Urine	Dog	Mecklenburg-West Pomerania
7689	15	6	2	26	10	279	4	*K. pneumoniae*	Feces	Horse	North Rhine-Westphalia
7697	2	5	1	1	9	1	501	*K. pneumoniae*	Feces	Dog	Bavaria
7698	3	5	2	1	16	1	363	*K. pneumoniae*	Uterus	Horse	Saxony

## Data Availability

All data are provided in the manuscript.

## Abbreviations

The following abbreviations are used in this manuscript:

*K.*	*Klebsiella*
WGS	Whole genomic sequencing
MLST	Multilocus sequence typing
ST	Sequence type
MDR	Multidrug-resistant
NJ	Neighbor-Joining
